# Getting old beats the alternative

**DOI:** 10.1186/s40695-021-00072-5

**Published:** 2022-03-02

**Authors:** Allison Kessler

**Affiliations:** 1 Division of Spinal Cord Injury Medicine, Shirley Ryan Abilitylab, 355 E Erie St, Chicago, IL 60611 USA; 2 Northwestern Feinberg School of Medicine, 420 E Superior St, Chicago, IL 60611 USA

**Keywords:** Aging, Paraplegia, Spinal cord injury, Disability

## Abstract

Getting old is hard, but it beats the alternative. This commentary explores some of the challenges of aging with a physical disability and the considerations taken to prevent further functional decline or injury during the aging process.

## Background

Aging can be difficult for any individual but when an individual has a physical disability, the aging process may present additional challenges, which may affect all aspects of live including basic activities of daily living. According to a recent report, 10.6% of the non-institutionalized working-age population (age 21–64) in the Unites States identify as having a disability. In this age group, the highest prevalence rate was for an “Ambulatory Disability” at 5.2% of the population [1]. This commentary explores some of the challenges faced by an individual in this mid-life age group aging with an ambulatory disability as well as the considerations taken to prevent further functional decline or injury during the aging process.

## Main text

Getting old is hard, but it beats the alternative. Over the years, I have made that joke many times when friends, family, or patients have told me they are tired of getting old, and while they generally agree with me, most also acknowledge that aging is a difficult process. Only more recently, as I entered my 30s, have I even begun to understand more fundamentally how aging changes the way we lead our lives. I have also heard my three older brothers, all in their 40s, and my 70+ year-old parents lament that they cannot do the things they used to and do not ‘bounce back’ in the same way. Generally the older we get, the harder it becomes to do things and the longer recovery becomes!

Having a spinal cord injury comes with its own set of challenges. I was 15 when I had my injury, and being young and healthy with a low BMI helped me tremendously with my physical rehabilitation process and ‘returning to my life’. I learned to adapt to my injury and found new ways of doing things. Looking back on the rest of high school and college, I am sometimes amazed at the things I put my body through and that it was able to tolerate all of it. Not only that but I realized that in trying to transition back to my boarding school life, I coped by hiding or minimizing my disability as much as possible. At the time, I felt the need to do this in order to be accepted by my peers without being excluded or treated differently.

This often translated behaviorally into not asking for help, accommodations, or adjustments to anyone else’s routine, lest I burden them. I was able to do this because of my health and youth. If I am honest, if my injury occurred today, I would likely have a much longer recovery process. I weigh more, I am not as strong, I have painful areas, I am less flexible, and my skin is thinner/more fragile.

I carried this laissez-faire attitude with me all the way through medical school, when things finally began to change. I began to understand that the world didn’t know or always care what I needed and that the only way I would get it would be to speak up for myself. In the final year of medical school, our student council planned a graduation event at a bar, which was not at all wheelchair accessible. When I requested a change of venue to allow me easier access, the coordinator was dismissive. He stated that I should just “adapt” as I had often done in the past, without making them change the venue for everyone else. To say that I was hurt and frustrated is an understatement. It took effort on my part to ask for an accommodation in order to both protect my physical wellbeing but also emotionally allow me to participate with my peers. That my colleagues, soon-to-be doctors, could not understand that what they were asking was both physically and emotionally harmful for someone with physical limitations was a revelation.

While everyone has his or her own challenges with aging, aging with a disability is harder. Our threshold is lower. On a good day, with the use of my wheelchair, shower chair and an accessible environment, I am completely independent and able to go about my daily life. However, a change that might make something a little more difficult for an able-bodied person can make an activity completely impossible for me.

I had my first child 2–1/2 years ago and the third trimester was incredibly difficult. I was unable to transfer into bed myself and I had to have my husband remove the box spring to lower the mattress or help me in. I was unable to transfer into the car by myself, which meant having to plan any trip or errand around when someone was available to help. After my daughter was born, I had a whole host of challenges trying to find ways to care for her safely with my limited mobility including adapting her sleeping and diaper changing areas and experimenting with various baby-carrying apparatuses. I also found positioning her for breast-feeding while also adequately taking pressure breaks to prevent skin-breakdown nearly impossible. Being a new parents is always a stressful time but I had to also take into account my own mobility and medical needs in coming up with a parenting plan for her.

More recently, I fell out of my wheelchair trying to reach something under a bed and fractured my leg. This was not the first or even 10th time I have fallen; 19 years ago it would have been fine. Instead, thanks to disuse osteopenia and age, my leg snapped. Further, how to best cast it became a challenge. The ‘textbook’ answer would have been to put the leg in extension but that would have made me incapable of doing most regular activities, including going to work. Ultimately, I had the leg splinted in 90 degrees of flexion to allow for appropriate positioning in the wheelchair. Despite this, I had to rely on others for certain activities during the next 8 weeks; I was initially unable to perform transfers, showering, or lower body dressing on my own. These are tasks that most people take for granted, and that I had taken for granted as activities that I had already ‘mastered’ despite my paraplegia; now I found myself having to relearn them again with an even bigger set of challenges. I have seen this time and again with some of my patients as well – they are used to doing some activity in a certain way and now, because of new illness, injury, or just age, they are no longer able to do those functions in the same manner. It is particularly challenging to explain why they can’t, or shouldn’t, continue to do things in the way they had found to cope with their disability, as I am not only asking them to change their habits but sometimes I am also asking them to re-examine their own disabled identity. It may seem to others that help is help, but I can tell you from both personal and professional experience that asking someone to accept help for something they did not need it for previously can be emotionally difficult – and for some untenable.

I recently read an op-ed on spinal cord injury (SCI) and burnout [2] where someone discussed their own SCI in terms of costing them 30 h per week of increased time to ‘manage’ their disability – something that is equivalent to a full time job. Add to that my actual full time job as a physician as well as my other full time job of being a mother (and wife) I have to acknowledge that there are only so many hours in the day. Some days it seems impossible to be able to ‘have it all’ or to ‘lean in’, as Sheryl Sandberg promotes [3]. Everyone my age seems to talk a lot about work/life balance but I am not sure anyone has perfected it, nor do I think that they are mutually exclusive. My work is part of my life and I intend to enjoy all of it as much as possible. To do that though I have learned that sometimes concessions have to be made. In order to maximize time with my daughter I have to be healthy – when I had a broken leg, I was unable to do some of the things with her I enjoy most. Rushing to ‘get things done’ ultimately resulted in a broken leg that cost me invaluable time with her. Being healthy becomes harder as time goes on though and is further challenging because of my underlying mobility issues. I have also had to acknowledge that things will take longer now than they did previously and not try to cut corners to make it like it ‘was before’. I have had to learn to outsource where possible, ask for help, budget extra time, multi-task, but perhaps more importantly, to prioritize. I more than likely will have to continue to adjust how I do things the older I get as I know that more limitations and challenges will come due to the natural aging process. I do not look forward to rotator cuff injuries that I am sure will plague me eventually but this realization of what might come has also allowed me to adjust what I am doing now in order to prevent future illness or injury as much as possible.

## Conclusions

Aging is not for the faint of heart, but I still recommend it over the alternative. Although it is sometimes hard as a physician to admit my limitations, if I, as a physician living with a spinal cord injury taking care of others with spinal cord injuries cannot ask for help or ask others to change environments or practices, who will do that for my patients? How can I expect my patients to protect themselves or to integrate back into their lives? Removing physical barriers and creating wheelchair access improves community, interpersonal, and professional reintegration for people with lower extremity mobility limitations. By speaking up about my needs, perhaps I can help to reduce stigma and improve access for the patients I care for on a daily basis. I certainly feel that my own personal growth has helped me connect and empathize with my patients more and has helped me better guide them through the aging process with the changes that it may bring. Although I know that aging will bring more challenges my way, I for one, am looking forward to the next stages of life.

## Data Availability

Not applicable.

## Abbreviation

SCI: Spinal cord injury
